# Immediate and delayed psychological effects of province-wide lockdown and personal quarantine during the COVID-19 outbreak in China

**DOI:** 10.1017/S0033291720003116

**Published:** 2020-08-13

**Authors:** Yiqun Gan, Jinjin Ma, Jianhui Wu, Yidi Chen, Huanya Zhu, Brian J. Hall

**Affiliations:** 1 School of Psychological and Cognitive Sciences and Beijing Key Laboratory of Behavior and Mental Health, Peking University, Beijing, People's Republic of China; 2 School of Psychology, Shenzhen University, Shenzhen, People's Republic of China; 3 Global and Community Mental Health Research Group, New York University (Shanghai), Shanghai, People's Republic of China; 4 Health, Behavior, and Society, Johns Hopkins Bloomberg School of Public Health, Baltimore, MD, USA

**Keywords:** COVID-19 outbreak, personal quarantine, province-wide lockdown, psychological distress, self-stigma

## Abstract

**Background:**

The COVID-19 pandemic has greatly affected public health and wellbeing. In response to the pandemic threat of the coronavirus epidemic, several countries, including China, adopted lockdown and quarantine policies, which may cause psychological distress. This study aimed to explore the psychological impact of province-wide lockdown and personal quarantine during the COVID-19 outbreak in China as well as the corresponding risk factors and protective factors.

**Methods:**

We examined the immediate (2-week) and delayed (2-month) impact of province-wide lockdown and personal quarantine on psychological distress in a national sample of 1390 Chinese residents.

**Results:**

No immediate impact of province-wide lockdown on psychological distress was observed, whereas personal quarantine increased individuals’ anxiety, fear, and anger. Despite the lack of initial association, psychological distress increased among those in province-wide lockdown. Self-stigma and personal control both significantly moderated the association between lockdown and psychological distress, but in different directions. Those with higher self-stigma and lower personal control were more impacted by the lockdown. Government support moderated the impact of quarantine on psychological distress, but not that of lockdown.

**Conclusions:**

The delayed effects of lockdown and quarantine on psychological distress were observed, and self-stigma, social support, and perceived control moderate the relationships. This study is the first to demonstrate the psychological costs of province-wide lockdowns on individuals’ mental health, providing evidence of the need for mitigation strategies and timely public mental health preparedness in countries with recent outbreaks of COVID-19.

## Introduction

In December 2019, coronavirus disease (COVID-19) emerged in Wuhan, Hubei Province, China and rapidly spread across China and into other countries, with over 1.9 million people infected and over 115 000 deaths to date. On 23 January 2020, Hubei Province – the epicenter of the novel coronavirus outbreak and home to 59 million people – was ‘locked down’. All buses, subways, ferries, and long-distance transport were suspended in the city. Citizens were not allowed to leave Hubei, and the airport and railway stations were closed. In provinces other than Hubei, there were different restriction regulations such as close down of highways and hotels, among some were especially to those from Hubei provinces.

In response to the announcement on 11 March 2020 by the World Health Organization (WHO) that the coronavirus epidemic was a pandemic threat, several other countries initiated their own lockdowns. This unprecedented public health emergency response delayed the growth and limited the magnitude of the COVID-19 epidemic in China, averting thousands of cases by 19 February (Tian et al., 2020), which limited the total number of cases to roughly 80 000 people. Despite the success of this action, the impact on the mental health and wellbeing of the general population needs to be quantified. The mental health of the general population during the COVID-19 epidemic has been investigated extensively. In the early stages of the outbreak in China, more than half of the participants rated the psychological impact as moderate-to-severe, and about one-third reported moderate-to-severe anxiety (Wang et al., 2020*b*). After 4 weeks, stress, anxiety, and depression still remained at the same level (Wang et al., 2020*c*). However, the impact of lockdown and quarantine on people's mental health remains unclear. This study is the first to assess the psychological consequences of province-wide lockdown and quarantine due to the COVID-19 pandemic.

### The psychological effects of personal quarantine

In addition to province-wide lockdowns in Hubei, China adopted a series of public health emergency measures including social distancing and personal quarantine to stop the spread of the virus. Here, ‘lockdown’ includes all people living in Hubei during the lockdown period, whereas ‘quarantine’ refers to mandatory quarantine for patients who had COVID-19, patients showing mild symptoms, those who had close contact with infected persons, or those who had recently traveled to Hubei Province. Between lockdown and personal quarantine, we speculated that personal quarantine would have larger effects on the mental health of the general population, since is it is a stricter measure and limits peoples’ freedom to a great extent. Although being quarantined, people were unable to leave their apartment buildings and apartment compounds, and in some cases, they were confined to their apartments. Relative to what is known about the impact of quarantine on mental health, no known study has evaluated the effect of citywide lockdowns.

These measures, although necessary for reducing the spread of the virus, are likely associated with psychological distress. A rapid review of 24 studies documenting reactions to quarantine suggested that negative psychological effects could include post-traumatic stress disorder (PTSD), anxiety, and depression (Brooks et al., 2020). People who were quarantined because of being in close contact with those who possibly had SARS reported various negative stress symptoms: 20% reported fear, 18% reported nervousness, 18% reported sadness, and 10% reported guilt (Reynolds et al., 2008). In Toronto, Canada, during the SARS outbreak, PTSD was found in 28.9% and depression in 31.2% of the 129 quarantined individuals, and stress and anxiety were commonplace among SARS-infected individuals (Maunder et al., 2003). Quarantined hospital staff were significantly more likely to report exhaustion, detachment from others, anxiety when dealing with febrile patients, irritability, insomnia, poor concentration, and indecisiveness (Bai et al., 2004). Stress responses during quarantine predict long-term anxiety and depression for up to a decade, demonstrating the expected impact of COVID-19 on public mental health (Charles, Piazza, Mogle, Sliwinski, & Almeida, 2013; O'Neill, Cohen, Tolpin, & Cimbolic Gunthert, 2004; Parrish, Cohen, & Laijrenceaij, 2011).

In the latest research on COVID-19, a series of cross-group studies found anxiety, depression, stress, insomnia, and PTSD symptoms to be at the middle-high level during the epidemic in China among the general public (Wang et al., 2020b), psychiatric patients (Hao et al., 2020), and healthcare workers (Tan et al., 2020*a*). Negative symptoms in psychiatric patients were significantly higher than in the general public (Hao et al., 2020), and a high correlation between psychological symptoms and physical symptoms in healthcare workers was found (Chew et al., 2020). In addition, one study on workers who returned to work found that, compared to ordinary people, there was a decrease in negative psychological effects when they returned to work, which illustrates the adverse effects of quarantine on mental health (Tan et al., 2020*b*).

### Correlates of psychological distress during quarantine

Public stigma is a principal risk factor for poor mental health among individuals in quarantine (Douglas, Douglas, Harrigan, & Douglas, 2009). Individuals in quarantine are significantly more likely to report social stigma and rejection from people in their local neighborhoods (Brooks et al., 2020). In China, stigma related to COVID-19 was quickly associated with people from Hubei, and they became known as virus carriers (Liu, Pei, & Xu, 2020). Stigma is particularly troubling, with respect to the likelihood of being denied social acceptance, tolerance, and allocation of societal resources (Chung & Slater, 2013). The social marginalization and ostracism associated with stigma can also harm emotional and physical health (Goffman, 1963; Mullen & Smyth, 2004; Twenge & Crocker, 2002). With the rapid spread of COVID-19, quarantined individuals not only are at risk of infection but also experience unfair treatment and rejection from individuals not in quarantine, which may lead to self-stigma. Individuals with internalized stigma subjectively generate self-bias because they belong to a group degraded by the public, resulting in negative emotions, especially inferiority, self-shame, and self-blame (Oexle et al., 2017).

Perceived self-control is an important factor in determining psychological state under stressful situations (Grote, Bledsoe, Larkin, Lemay, & Brown, 2007) and is generally considered to be a protective factor for various psychiatric diseases (Kadden & Litt, 2011). The theory of locus of control refers to individuals who believe that their self-behavior is controllable are more inclined to actively cope with the stressful environment, which enhances their likelihood of attaining successful outcomes, and can thus achieve a good emotional state under stress (Lefcourt, 2014; Li et al., 2016; Skinner, 1995). Results from neuroscience research also indicate that internal control can stimulate positive emotions, and this function engages brain networks processing self-referential information in the cortical midline, which is related to activity in the ventromedial prefrontal cortex (Stolz, Müller-Pinzler, Krach, & Paulus, 2020).

At present, one of the main theories about the role of social support in the relationship between stress and psychological outcomes is the stress buffering model. According to the stress buffering model, social support can buffer or regulate the negative impact of emergencies on mental health, and social support is a regulatory factor in the relationship between stress and psychological distress. Cobb (1976) suggested that social support can help individuals to cope with crises and better adapt to the changes of the environment, and the effect of social support on mental health can only occur when there is a sudden crisis. According to the buffering model, social support can improve an individual's sense of security and self-confidence in the face of stress by providing the relevant social or psychological resources for the individual. Some scholars also believe that the ‘main effect model’ and ‘buffer model’ of social support complement each other. The social support obtained by individuals can not only produce the main effect on depressive symptoms by improving self-confidence or self-control, but also produce the buffer effect on depressive symptoms under pressure (George, 1989).

In public health emergency and response, the role of social support is widely noted by researchers. Support available from the government may refer to policy, capital, public opinion, and other aspects perceived by individuals as support (Huang, Tan, & Liu, 2016). According to the theory of resource conservation (Hobfoll, Halbesleben, Neveu, & Westman, 2018), macro government support, meso social support, and micro sense of control are all core values for people, and they are important resources that affect mental health. Therefore, the roles of perceived social support and government support are likely to protect mental health of the general population during the COVID-19 quarantine and lockdown.

### Objectives and hypotheses

The current study examined the immediate (2 weeks) and delayed (2 months) psychological impact of the province-wide lockdown and personal quarantine during the COVID-19 outbreak in China. The key factors associated with lockdown, quarantine, and mental health, including self-stigma, perceived control, and social support, were examined. In particular, we proposed the following hypotheses:

H1: Province-wide lockdown and personal quarantine are associated with depression, fear, anxiety, and anger.

H2: Psychological distress due to province-wide lockdown and personal quarantine increases over time.

H3: Self-stigma has both a main effect and a moderating effect on psychological distress among individuals in lockdown and personal quarantine.

H4: Internal psychological resources (e.g. perceived control) and external psychological resources (e.g. social support and governmental support) both have a main effect and a moderating effect on the relationship of province-wide lockdown and personal quarantine with psychological distress.

H5: Lockdown and external psychological resources will interact to predict psychological distress. Those under lockdown who possess low levels of social support and high levels of self-stigma will be the group most vulnerable to psychological distress.

## Methods

### Participants

#### Recruitment

Data were collected across two time points. The baseline was conducted during the initial epidemic period (1 to 8 February 2020), and the follow-up was conducted from 17 to 24 March 2020, which was considered the recovery period for COVID-19 in China, 14 days before the Hubei Province lockdown ended. Self-reported questionnaires were administered to participants through a survey website hosted by WenJuanXing (Changsha Haoxing Information Technology Co., Ltd., China). Questionnaire recruitment included commissioning WenJuanXing to invite users to participate on its professional questionnaire platform. Six filler items (e.g. ‘I usually feel that winter is hotter than summer’) were included to exclude invalid responses and ensure data quality. The inclusion criteria were being a Chinese citizen, responding correctly to at least four filler items, and having completed junior high education or higher. After excluding invalid responses via the six filler items, baseline and follow-up data were matched according to WenJuanXing unique usernames. Ultimately, 1390 participants who completed the two surveys were included in the analyses.

At baseline, 5019 participants were contacted. According to the inclusion criteria, seven were deleted due to incompleteness and 1779 due to at least three wrong answers to filler items; and ultimately, 3233 responses were retained. The response rate was 64.4%.

Among the included participants, approximately 50% (*n* = 1630) were from Hubei and comprised the ‘lockdown sample’. After matching, the final analytic sample consisted of 1390 Chinese residents who participated in the follow-up. Approximately 25% (*n* = 403) were from Hubei Province. Participants from outside Hubei were distributed across the 29 provinces and regions in mainland China. Participants’ demographics are shown in Table 1. Among the 3233 participants, 10 were confirmed cases (three were in lockdown, seven were not in lockdown) and 55 were suspected cases (45 were in lockdown, 10 were not in lockdown) during the first round of our investigation, and 333 in total were quarantined. Participants who were suspected or confirmed cases all belonged to the quarantined group. The study procedures were approved by the first author's Institutional Review Board. All participants provided written informed consent.

**Table 1.** Sociodemographic characteristics of the sample

Characteristics	Category	Province lockdown (*N* = 403)	Province not lockdown (*N* = 987)
Gender	Male	166 (41.2%)	429 (43.5%)
	Female	237 (58.8%)	558 (56.5%)
Age (years) [mean (s.d.) (range)]		29.09 (9.05) (14–67)	31.38 (8.70) (16–65)
Education	Junior high school	13 (3.2%)	15 (1.5%)
	High school	45 (11.2%)	30 (3.0%)
	Junior college	84 (20.8%)	158 (16.0%)
	Bachelor degree	229 (55.8%)	698 (70.7%)
	Master degree or above	32 (7.9%)	86 (8.7%)
Subjective SES (rank 1–10 in total)	Rank 1–3	147 (36.5%)	175 (17.7%)
	Rank 4–7	248 (61.5%)	785 (79.5%)
	Rank 8–10	8 (2.0%)	27(2.7%)
Occupation	Full-time students	123 (30.5%)	172 (17.4%)
	National government personnel	7 (1.7%)	22 (2.2%)
	Employees of enterprises/institutions	165 (40.9%)	604 (61.2%)
	Professional technical staff	30 (7.4%)	66 (6.7%)
	Business/service personnel	27 (6.7%)	47 (4.8%)
	Medical industry personnel	7 (1.7%)	16 (1.6%)
	Education industry personnel	11 (2.7%)	32 (3.2%)
	Others	33 (8.2%)	28 (2.8%)
Health status	Diagnosed with COVID-19	3 (0.7%)	7 (0.7%)
	Suspected as having COVID-19	45(11.2%)	10 (1.0%)
	Quarantined for close contact	135 (33.5%)	133 (13.5%)
	Not infected	220 (54.6%)	837 (84.8%)

Values are numbers (percentages) unless stated otherwise.

### Instruments

*The Mental Health Response to Public Health Emergency Scale* (Tang et al., 2007) was used to measure emotional responses, including depression (e.g. ‘Less energy than before’), anxiety (e.g. ‘Experiencing increased heartbeat, sweating, and blushing’), fear (e.g. ‘Worrying that me and my family will be infected’), and anger (e.g. ‘The attitude of not paying attention to the epidemic makes me angry’). There are 25 items in total, with each item rated on a 4-point Likert type response scale (1 = ‘never’, 4 = ‘very often’). The Cronbach's *α* for the total scale at baseline and follow-up were 0.90 and 0.81 for depression, 0.65 and 0.67 for anxiety, 0.72 and 0.69 for fear, and 0.82 and 0.79 for anger, respectively.

*The PTSD Checklist-5* (*PCL-5*; Weathers et al., 2013) measured posttraumatic stress symptoms related to the COVID-19 outbreak. The index event was the COVID-19 epidemic, and symptoms were assessed at follow-up to adhere to the 1-month post event criterion for PTSD. The scale consists of four dimensions: intrusion symptoms (e.g. ‘Repeated, disturbing dreams of the stressful experience’), avoidance symptoms (e.g. ‘Avoiding memories, thoughts, or feelings related to the stressful experience’), alternations in cognition and emotion (e.g. ‘Trouble remembering important aspects of the stressful experience’), and arousal (e.g. ‘Feeling jumpy or easily startled’). There are 20 items in total, and each item is scored on a 4-point Likert-type scale (1 = ‘not at all’, 4 = ‘extremely’). The Cronbach's *α* for the scale was 0.93.

*The Perceived Stress Scale-10* (Cohen, Kamarck, & Mermelstein, 1983; Yang & Huang, 2003) includes 10 items assessing stress during the epidemic outbreak. An example item is ‘Have you felt unable to control the important things in your life during the outbreak?’ Each item is scored on a 5-point Likert scale (1 = ‘never’, 5 = ‘very often’). The Cronbach's *α* was 0.80 at baseline and follow-up.

*The Self-stigma Scale*, adapted from the Self-stigma Scale developed by Fife and Wright (2000), measures stigma against quarantined individuals due to confirmed or suspected infection. The scale includes four dimensions: social isolation (e.g. ‘I need the care and comfort of others more than during the non-outbreak period’), social rejection (e.g. ‘I don't get the respect I deserve as a non-quarantined person’), internalization shame (e.g. ‘I feel guilty for being quarantined for the outbreak’), and exposure (e.g. ‘I am afraid to tell others that I have been quarantined for the outbreak’). There are 13 items in total, with each item scored on a 4-point Likert scale (1 = ‘strongly disagree’, 4 = ‘strongly agree’). The Cronbach's *α* was 0.77 and 0.80 at baseline and follow-up, respectively.

*Perceived Social Support* from interpersonal relationships is measured using the Short Form Multidimensional Scale of Perceived Social Support (Porter et al., 2019). The scale specifically includes three dimensions: family support (e.g. ‘I get the emotional help and support I need from my family’), friends’ support (e.g. ‘I can talk about my problems with my friends’), and significant other's support (e.g. ‘There is a special person who is around when I am in need’). The scale consists of six items, which are scored on a 7-point Likert-type scale (1 = ‘strongly disagree’, 7 = ‘strongly agree’). The Cronbach's *α* was 0.80 and 0.82 at baseline and follow-up, respectively.

*Perceived Government Support*, adapted from the Multidimensional Scale of Perceived Social Support (Porter et al., 2019), aims to measure perceived support from the government (e.g. ‘Medical supplies provided by the government of your country can make the epidemic pass quickly’). It consists of five items, which are scored on a 7-point Likert scale (1 = ‘extremely disagree’, 7 = ‘extremely agree’). The Cronbach's *α* was 0.79 and 0.76 at baseline and follow-up, respectively.

*The Perceived Control Scale* (Pallant, 2000) measures the degree to which individuals feel they are in control of their internal states, including emotions (e.g. ‘I don't have much control over my emotional reaction to stressful situations’), thoughts (e.g. ‘I am usually able to keep my thoughts under control’), and physical reactions (e.g. ‘There is not much I can do to relax when I get uptight’). It consists of 18 items, which are scored on a 5-point Likert scale (1 = ‘very disagree’, 5 = ‘very agree’). The Cronbach's *α* was 0.93.

*Lockdown and quarantine*: Lockdown experience was measured by whether the respondent lived in Hubei Province using one item: ‘In which city do you currently live?’ Quarantine experience was also measured by one item: ‘Do you have any experience of being quarantined (including hospital quarantine, home quarantine, centralized observation isolation, etc.)? (*Note*: Uninfected people taking the initiative to reduce the number of times they go out, also called “self-isolation”, is not included)’.

At baseline, the Mental Health Response to Public Health Emergency Scale, the Perceived Stress Scale, the Self-stigma Scale, the Perceived Social Support and Perceived Government Support Scale, and the Perceived Control Scale were administered. At follow-up, all of the above scales were re-administered, and the PCL-5 was also included.

### Statistical analyses

Of the 1390 matched participants, 333 were quarantined, and 403 experienced province-wide lockdown. First, we compared the immediate effects of quarantine and lockdown on depression, anxiety, and fear at baseline by comparing groups who were in personal quarantine (*n* = 333 *v*. *n* = 1057) and province-wide lockdown (*n* = 403 *v*. *n* = 987). As previous studies suggested that economically disadvantaged groups are less likely to receive accurate information regarding COVID-19, protective equipment, and health services (Tran et al., 2020), we used subjective socioeconomic status (SES) as a covariate in all analyses.

Second, we examined the interaction between province-wide lockdown and personal quarantine on psychological distress (i.e. depression, anxiety, fear, and anger), with sex, age, education, subjective SES, and baseline outcome as covariates. We performed a multivariate analysis of covariance (MANCOVA) for these analyses, which allowed us to assess the outcomes simultaneously and control for the inflation of *p* values in multiple tests.

Finally, we evaluated whether self-stigma, social support, governmental support, and perceived control moderated the effect of lockdown on psychological distress (depression, anxiety, fear, anger, perceived stress, and PTSD symptoms). In addition, we examined the three-way interaction of lockdown, self-stigma, and social support on predicting psychological distress. We used the bootstrapping procedure by Preacher and Hayes (2008) and the corresponding SPSS macro Model 1 and Model 3. We set the number of samples at 5000 and used a bias correction option. To test the moderating effect of self-stigma (follow-up), we controlled for sex, age, education, and SES. To test the moderating effect of social support (follow-up), governmental support (follow-up), and perceived control (follow-up), we controlled for sex, age, education, SES, and psychological distress at T1 at baseline (Table 2).

**Table 2.** Descriptive analysis: means, and s.d., bivariate correlations

	*M*	s.d.	1	2	3	4	5	6	7	8	9	10	11	12	13	14	15
(1) Self-Stigma_T2	2.04	0.45	1.00														
(2) Social Support_T2	5.44	0.88	−0.12**	1.00													
(3) Government Support_T2	5.97	0.71	−0.18**	0.43**	1.00												
(4) Perceived Control_T2	3.63	0.63	−0.21**	0.47**	0.32**	1.00											
(5) Depression_T1	1.49	0.50	0.19**	−0.15**	−0.16**	−0.30**	1.00										
(6) Depression_T2	1.60	0.52	0.29**	−0.22**	−0.19**	−0.42**	0.56**	1.00									
(7) Anxiety_T1	1.31	0.38	0.23**	−0.14**	−0.19**	−0.27**	0.63**	0.44**	1.00								
(8) Anxiety_T2	1.35	0.39	0.37**	−0.21**	−0.25**	−0.35**	0.40**	0.64**	0.53**	1.00							
(9) Fear_T1	2.33	0.65	0.27**	0.00	−0.03	−0.16**	0.39**	0.27**	0.51**	0.36**	1.00						
(10) Fear_T2	2.22	0.60	0.35**	−0.01	−0.06*	−0.21**	0.29**	0.42**	0.40**	0.54**	0.60**	1.00					
(11) Angry_T1	3.08	0.84	0.09**	0.07**	0.10**	−0.04	0.15**	0.12**	0.19**	0.14**	0.40**	0.26**	1.00				
(12) Angry_T2	3.13	0.78	0.13**	0.08**	0.17**	−0.08**	0.12**	0.19**	0.16**	0.18**	0.34**	0.38**	0.43**	1.00			
(13) Perceived Stress_T1	2.49	0.60	0.23**	−0.18**	−0.17**	−0.41**	0.41**	0.34**	0.49**	0.35**	0.45**	0.35**	0.23**	0.23**	1.00		
(14) Perceived Stress_T2	2.36	0.54	0.39**	−0.30**	−0.26**	−0.54**	0.35**	0.50**	0.41**	0.53**	0.34**	0.45**	0.16**	0.23**	0.61**	1.00	
(15) PTSD Symptoms_T2	1.97	0.64	0.35**	−0.34**	−0.26**	−0.56**	0.44**	0.63**	0.51**	0.67**	0.38**	0.51**	0.17**	0.27**	0.44**	0.61**	1.00

*p* < 0.05; **p < 0.01.

## Results

### Attrition analyses

Attrition analyses for demographic and outcome variables were performed between people who participated in follow-up (*n* = 1390), and those who were lost to follow-up (*n* = 1843). Chi-square test results showed that more men (χ^2^ = 7.80, *p* = 0.005) and people outside Hubei Province (χ^2^ = 478.31, *p* < 0.001) were lost to follow-up. Older participants (*F*_1,3209_ = 25.58, *p* < 0.001, *η*_p_^2^ = 0.008), and those with greater psychological distress (Pillai's trace = 0.018, *F*_5,3227_ = 11.67, *p* < 0.001, *η*_p_^2^ = 0.018), including depression (*F*_1,3231_ = 43.19, *p* < 0.001, *η*_p_^2^ = 0.013), anxiety (*F*_1,3231_ = 24.51, *p* < 0.001, *η*_p_^2^ = 0.008), fear (*F*_1,3231_ = 18.55, *p* < 0.001, *η*_p_^2^ = 0.006), perceived stress (*F*_1,3231_ = 37.20, *p* < 0.001, *η*_p_^2^ = 0.011) were lost to follow-up.

### Immediate effects at baseline

The main effects of both personal quarantine (Pillai's trace = 0.011, *F*_4,1379_ = 3.81, *p* = 0.004, *η*_p_^2^ = 0.011) and lockdown (Pillai's trace = 0.013, *F*_4,1379_ = 4.44, *p* = 0.001, *η*_p_^2^ = 0.013) were significant. Univariate tests indicated that individuals in lockdown reported less fear (*F*_1,1382_ = 6.69, *p* = 0.010, *η*_p_^2^ = 0.005) than individuals not in lockdown. Further, the univariate tests indicated that individuals in quarantine reported greater anxiety (*F*_1,1382_ = 3.93, *p* = 0.048, *η*_p_^2^ = 0.003); fear (*F*_1,1382_ = 12.27, *p* < 0.001, *η*_p_^2^ = 0.009) and anger (*F*_1,1382_ = 6.65, *p* = 0.010, *η*_p_^2^ = 0.005) than individuals not in quarantine.

### Interaction effects of province-wide lockdown and personal quarantine on delayed psychological distress adjusting for baseline psychological distress

We performed a MANCOVA to examine the interaction between province-wide lockdown and personal quarantine on psychological distress (i.e. depression, anxiety, fear, and anger), with sex, age, education, subjective SES, and baseline outcome as covariates. Univariate tests indicated no significant interaction between lockdown and personal quarantine on depression, anxiety, fear, or anger.

The main effect of lockdown was significant for depression (*F*_1,1378_ = 5.51, *p* = 0.019, *η*_p_^2^ = 0.004) and for fear (*F*_1,1378_ = 9.06, *p* = 0.003, *η*_p_^2^ = 0.007), such that individuals in lockdown reported more depressed but reported less fear. The effects of lockdown on anxiety, and anger were not significant.

The main effect of personal quarantine on anxiety was significant (*F*_1,1378_ = 11.59, *p* = 0.001, *η*_p_^2^ = 0.008), such that individuals in personal quarantine reported higher anxiety, whereas main effects on depression, fear, and anger were nonsignificant.

### Interaction effect of province-wide lockdown, personal quarantine on follow-up self-stigma, social support, and perceived control adjusting for baseline measures

We performed a MANCOVA to examine the interaction between province-wide lockdown and personal quarantine on T2 self-stigma, social support, governmental support, and perceived control, with sex, age, education, subjective SES, and baseline outcome as covariates. No significant interaction was found between lockdown and personal quarantine: *F*_4,775_ = 1.40, *p* = 0.231, *η*_p_^2^ = 0.007. Univariate tests indicated the main effect of lockdown on self-stigma was significant (*F*_1,778_ = 4.78, *p* = 0.029, *η*_p_^2^ = 0.006), such that individuals in province lockdown reported lower self-stigma, but not for social support, governmental support, or perceived control. The main effect of personal quarantine on all the variables was non-significant.

### Moderating effect of self-stigma, support, and perceived control

Self-stigma moderated the effect of province-wide lockdown on depression [Δ*R*^2^ = 0.0040, *B* = 0.16, *F*_1,1168_ = 5.52, 95% confidence interval (CI) = 0.02–0.30, *p* = 0.020]; anxiety (Δ*R*^2^ = 0.0042, *B* = 0.12, *F*_1,1168_ = 5.71, 95% CI = 0.02–0.22, *p* = 0.017); perceived stress (Δ*R*^2^ = 0.0119, *B* = 0.29, *F*_1,1168_ = 17.37, 95% CI = 0.15–0.42, *p* < 0.001); and PTSD symptoms (Δ*R*^2^ = 0.0058, *B* = 0.23, *F*_1,1168_ = 8.05, 95% CI = 0.07–0.40, *p* = 0.005).

**Fig. 1:**
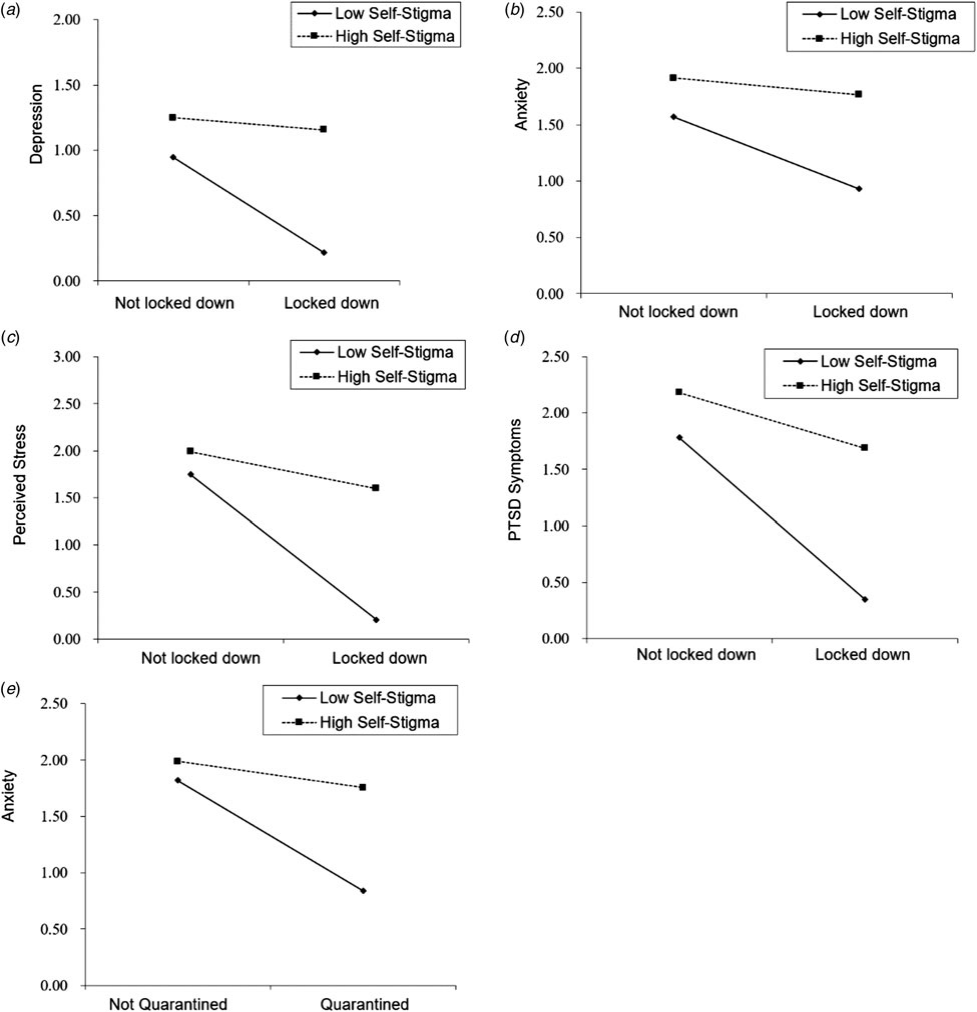
Moderate effects of self-stigma between province lockdown/personal quarantine and psychological distress. (*a*) Moderate effects of self-stigma between province lockdown and depression; (*b*) moderate effects of self-stigma between province lockdown and anxiety; (*c*) moderate effects of self-stigma between province lockdown and perceived stress; (*d*) moderate effects of self-stigma between province lockdown and PTSD symptoms; and (*e*) moderate effects of self-stigma between personal quarantine and anxiety.

A simple slope test with +1s.d. and −1s.d. group of self-stigma showed that for individuals with a low level of self-stigma, the lockdown did not affect their levels of depression, anxiety, or perceived stress, whereas for individuals with a high level of self-stigma, the lockdown was associated with greater depression (*t* = 4.20, *p* < 0.001), anxiety (*t* = 3.22, *p* = 0.001), and perceived stress (*t* = 5.03, *p* < 0.001) (Fig. 1*a*–*c*). For PTSD symptoms, the lockdown had no effect on individuals with a high level of self-stigma, whereas the lockdown was associated with decreased PTSD symptoms among individuals with a low level of self-stigma (*t* = −2.15, *p* = 0.321) (Fig. 1*d*).

Regarding personal quarantine, a significant moderating effect was only found for anxiety symptoms (Δ*R*^2^ = 0.0091, *B* = 0.19, *F*_1,1168_ = 12.73, 95% CI = 0.08–0.29, *p* = 0.004). A simple slope test demonstrated that for those with a low level of self-stigma, personal quarantine was not associated with anxiety, whereas for individuals with a high level of self-stigma, personal quarantine was associated with greater anxiety (*t* = 4.88, *p* < 0.001) (Fig. 1*e*).

**Fig. 2:**
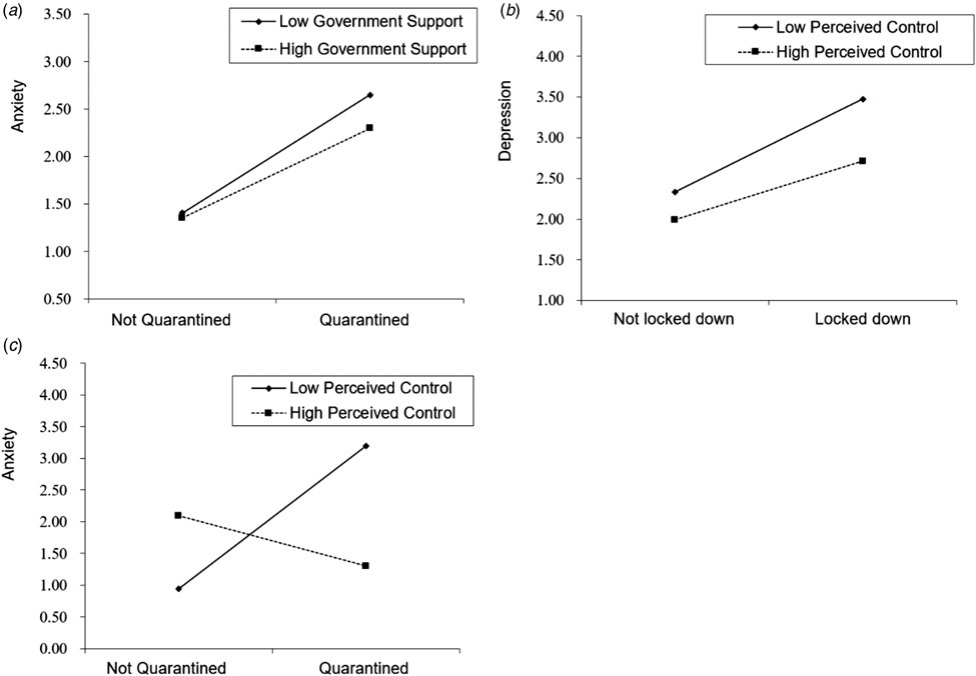
Moderate effects of government support and perceived control between province lockdown/personal quarantine and psychological distress. (*a*) Moderate effects of government support between personal quarantine and anxiety; (*b*) moderate effects of perceived control between province lockdown and depression; and (*c*) moderate effects of perceived control between personal quarantine and anxiety.

Social support did not moderate the effect of province-wide lockdown or personal quarantine on psychological distress. Governmental support did not moderate the effect of province-wide lockdown on psychological distress. Regarding personal quarantine, governmental support moderated the effect of quarantine on anxiety (Δ*R*^2^ = 0.0036, *B* = −0.074, *F*_1,1381_ = 5.38, 95% CI = −0.14 to −0.01, *p* = 0.020). A simple slope test demonstrated that for individuals with a high level of governmental support, personal quarantine was not associated with anxiety, whereas for individuals with low levels of perceived governmental support, personal quarantine was associated with greater anxiety symptoms (*t* = 4.71, *p* < 0.001) (Fig. 2*a*).

Perceived control moderated the effect of province-wide lockdown on depression (Δ*R*^2^ = 0.0034, *B* = −0.11, *F*_1,1381_ = 5.76, 95% CI = −0.19 to −0.02, *p* = 0.017), but not on anxiety, perceived stress, or PTSD symptoms. A simple slope test demonstrated that for individuals with high levels of perceived control, province-wide lockdown was not associated with depression, whereas for individuals with low levels of perceived control, province-wide lockdown was associated with increased depression (*t* = 3.88, *p* < 0.001) (Fig. 2*b*).

Perceived control moderated the effect of personal quarantine on anxiety (Δ*R*^2^ = 0.0028, *B* = 0.02, *F*_1,1381_ = 4.55, 95% CI = 0.01–0.03, *p* = 0.033), but not depression, perceived stress, or PTSD symptoms. A simple slope test demonstrated that for individuals with high levels of perceived control, personal quarantine was not associated with anxiety, whereas for individuals with low levels of perceived control, province-wide lockdown was associated with increased anxiety (*t* = 4.42, *p* < 0.001) (Fig. 2*c*).

**Fig. 3:**
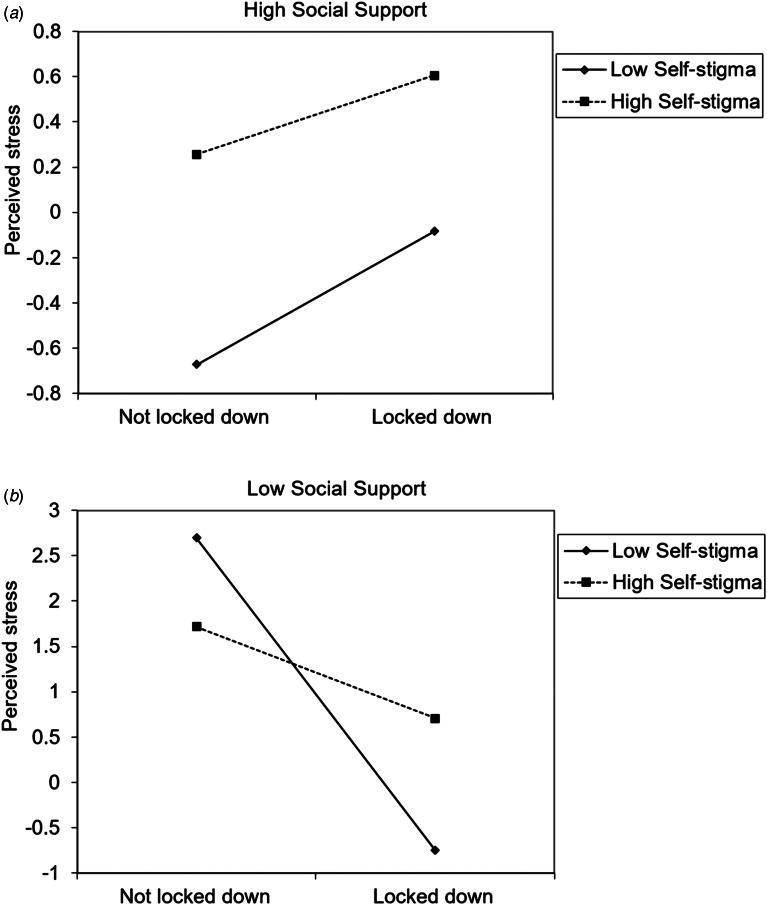
Interaction effect of social support, self-stigma, province lockdown on perceived stress.

We used Model 3 in SPSS Process macro to examine the three-way interaction of social support × self-stigma × province-wide lockdown on four psychological distress measures and found this three-way interaction was significant for perceived stress (Δ*R*^2^ = 0.0047, *B* = −0.19, *F*_1,1164_ = 7.48, 95% CI = −0.32 to −0.05, *p* = 0.006) (Fig. 3) but not for the other three measures. Simple main effect analysis showed that for those not in lockdown, social support did not modify the relationship of self-stigma on perceived stress; in contrast, for those in lockdown, social support modified the relationship of self-stigma on perceived stress. For people with high levels of self-stigma, lockdown was significantly associated with high perceived stress for people with low (*β* = 0.20, *t* = 4.37, *p* < 0.001) or middle social support (*β* = 0.10, *t* = 2.78, *p* = 0.006), but not significant for people with high social support (*β* = 0.01). For people with low or middle levels of self-stigma, lockdown was not significant associated with perceived stress regardless of the level of social support.

## Discussion

In a national sample utilizing a longitudinal follow-up design, no immediate impact of province-wide lockdown on psychological distress was observed, whereas personal quarantine increased individuals’ anxiety, fear, and anger. Despite the lack of initial association, psychological distress increased among those in province-wide lockdown. Self-stigma and personal control significantly moderated the effect of lockdown on psychological distress, but in different directions. Those with higher self-stigma and lower personal control were more vulnerable to lockdown. Social support did not modify the effect of lockdown or quarantine on psychological distress. This is the first study to examine the effects of public health strategies for COVID-19 in China.

### The psychological typhoon eye effect

At baseline, province-wide lockdown was not associated with psychological distress, although as predicted, significant negative effects on depression, anxiety, and fear were observed in quarantined individuals. During the province-wide lockdown, there was no significant indication of more distress at baseline, and notably, decreased levels of fear were observed. Wang et al. focused on the relationship between the duration of time spent at home and anxiety, depression, and stress at the beginning and peak of the outbreak and found that the duration of time at home did not correlate with anxiety, depression, stress, or PTSD symptoms at 4 weeks after the beginning of the COVID-19 outbreak. Our study also found that lockdown was not associated with distress at baseline (Wang et al., 2020*b*).

This could be explained by the ‘psychological typhoon eye effect’, which was proposed to describe the psychological response to a disaster, borrowing the phenomenon of ‘typhoon eye’ from meteorology, wherein the air around a typhoon rotates violently, whereas the air inside is relatively weak. Similarly, the closer the time period is to the high-risk stage, the calmer individuals are (Li et al., 2009; Liang et al., 2008; Lindell & Earle, 1983; Maderthaner, Guttmann, Swaton, & Otway, 1978). A similar phenomenon was observed during the SARS epidemic in Hong Kong, during which anxiety among residents in epidemic areas was lower than that of residents in non-epidemic areas (Xie, Xie, Rui, & Zhang, 2003).

This counterintuitive phenomenon can be understood mainly through two theories: first, the cognitive dissonance theory (Cooper, 2007; Festinger, 1962), that is, Hubei residents living in the most serious epidemic areas experienced cognitive dissonance because of the objective fact that the most serious epidemic situation in their living areas cannot be changed or controlled. To alter this cognitive dissonance, Hubei residents may have reduced their threat perception of the virus. Second, this phenomenon can be explained by the mere exposure effect, which suggests that compared to residents in a non-disaster area or light disaster area, residents in a heavy disaster area gradually adapt to and become accustomed to the environment due to long-term exposure to the high-risk environment, thus affecting the judgment of their own risk level (Xu et al., 2020).

Zheng et al. (2015) noted that the phenomenon of psychological typhoon eye effect aids in the prediction of the public's general response after an emergency, and investigators are advised to be cautious when interviewing survivors and evaluating post-disaster assistance; the information provided by the victims may be affected by this phenomenon. In this scenario, or in any other surveys that rely on self-report, we should be fully aware of the possibility that the negative psychological effects of province-wide lockdown could be moderated or biased by the psychological typhoon eye effect and thus make judgments taking measures from all results into consideration.

### Delayed psychological consequences of province-wide lockdown

Of the four psychological consequence indicators in the study follow-up from 17 to 24 February 2020, three appeared to be significant. Still affected by the psychological typhoon eye effect, the locked-down individuals reported significantly less fear. However, it was notable that significantly higher levels of depression were observed among those who were locked down. As mentioned above, the psychological consequence of province-wide lockdown has never been documented before, as no such event has occurred in modern history. The control of baseline outcome variables provides some clues to interpret the results, as there are marked individual differences in how they respond to such events over time. A growing body of longitudinal studies indicates that the majority of individuals’ long-term psychological reactions to an epidemic can be reliably captured by four prototypical outcome patterns or trajectories across time (Bonanno, 2004, 2005). In this study, however, we could not identify the pattern of each trajectory due to limited follow-up time, although a global trend of deteriorated reactions was found.

The results showed that the province-wide lockdown, in fact, had a long-term psychological impact on the 59 million individuals who remained in Hubei. For them, freedom was limited, medical resources were lacking, and their belief in the basic security of life was affected for a certain period of time (Wang et al., 2020*a*). However, management of large-scale infectious diseases often entails the temporary sacrifice of citizens’ freedom and civil rights. Similar to Bonanno et al.'s (2008) study of SARS in Hong Kong, people in Hubei did not know how long the epidemic and lockdown would last. Such uncertainty may have influenced the increase in the levels of depression and perceived stress observed in this study. Other possible factors include media coverage and misinformation about the COVID-19 outbreak.

### Factors that exacerbated the effects of province-wide lockdown and personal quarantine

Similar to many epidemic studies, the most significant risk factor that exacerbated the negative psychological effects of province-wide lockdown is self-stigma, as seen in the significant moderating effects of self-stigma on all four indicators: depression, anxiety, perceived stress, and PTSD symptoms. Self-stigma was also a risk factor in personal quarantine; however, this only affected anxiety. The results, in fact, highlighted the region-related self-stigma against residents of Hubei – the epicenter of the epidemic outbreak in China.

The history of infectious diseases highlights that individuals unconsciously discriminate against and exclude those who are isolated. The fact that the virus can be asymptomatically carried indirectly is a social problem of self-stigmatization and shame, as has been seen in previous epidemics (Pappas, Kiriaze, Giannakis, & Falagas, 2009). In addition, during the outbreak of SARS in Canada in 2003, the public avoided and stigmatized the infected and the location of the original infection and withdrew their social support (Pappas et al., 2009). In this COVID-19 outbreak, isolation of infected groups associated with Hubei – although it may, in fact, have had a positive effect on the prevention of the spread of the virus to a certain extent – also reduced the perceived social support of infected groups and caused them to be stigmatized. There are various methods of stigmatization, for example, avoiding the license plate number beginning with ‘E’ (Hubei) and refusing hotel accommodation to those from Hubei (Lin et al., 2020). These practices may be effective at controlling the spread of the epidemic for a certain period of time but are also suspected of unwittingly encouraging discrimination and exclusion, and these may have negatively affected the people of Hubei Province.

Previous studies have shown that there are two levels of cushions or psychological resources that can buffer the negative psychological consequences and protect individuals experiencing epidemic stress. The first level of protection is social support, including care and support from society, work, families, and organizations, among others. Due to its unique role, we measured governmental support separately as a unique type of social support. A previous study suggested that understanding social support is important for understanding and predicting individual mental health (Sen, Aguilar, & Goldbach, 2010). Our results support the main effect model of social support, as indicated by the significant correlations between social and governmental support on the one hand and depression, anxiety, fear, and PTSD symptoms on the other. Previous research also found that the average PTSD score of those who did not return to work during the epidemic mitigation period was three times that of those who returned to work, which may be related to high social support and self-control in the work environment (Tan et al., 2020*a*, *b*).

Only governmental support, which is found to have increased during the two time points, demonstrated a buffering effect in protecting quarantined individuals from anxiety. In general, the results demonstrate that support from the Chinese government for those in quarantine or lockdown played an increasingly important role in protecting individuals’ mental health.

The current study found that there was a significant negative correlation between discrimination perception and social support. This suggests that the decrease of social support partially explains the threat effect of discrimination perception on mental health, thus supporting the threat model of social support. According to the stress coping theory (Lazarus & Folkman, 1984), discrimination is a significant source of stress for members of vulnerable groups, and perceived risk information puts individuals in a state of stress.

This study was the first to identify a three-way interaction among external resource factors. In particular, we found individuals in lockdown possessing low levels of social support and high levels of self-stigma were the most vulnerable to experiencing perceived stress. High self-stigma is an important risk factor for perceived stress, but in our study, as social support improved, the positive correlation between lockdown and perceived stress gradually weakened, thereby providing a buffer to the psychological distress caused by self-stigma during the lockdown period. This result suggests target groups for possible psychological intervention for people experiencing lockdown. In addition, reducing self-stigma and enhancing social support might be important for people in lockdown. In particular, self-affirmation training (Schmeichel & Vohs, 2009; Sherman & Cohen, 2006) in WeChat groups could be a promising online intervention for those in lockdown.

The results of this study point to several key practical suggestions. First, the mass media could be used to reduce stigma and discrimination against individuals in lockdown. Second, the community and society should take effective measures to help people in lockdown by establishing or improving the social support system, which is conducive to reducing the impact on psychological health. Third, psychological public health workers could help individuals enhance their coping skills, including information regarding epidemic-related knowledge (Tran et al., 2020), identification of high-risk groups (Ho, Chee, & Ho, 2020), and providing professional psychological intervention services (Duan & Zhu, 2020). They may also be able to help reduce the pressure of discrimination and enhance individuals’ sense of self-control to better manage the adverse effects of lockdown.

### Limitations

This is the first study in China to explore the psychological consequences of lockdown and quarantine using a large sample size and follow-up. Despite these strengths, there are several limitations. First, the attrition rate was high from the first wave to second wave: attrition analyses did find some differences between groups, which may introduce selection bias. However, the differences in populations were negligible as evidenced by small effect size differences. Second, some questionnaires were not administered at both time points. Third, due to the two time points, we could not examine psychological outcome trajectories using latent growth curves. The epidemic of COVID-19 is still ongoing both in China and globally; the long-lasting psychological consequences still need to be investigated. Last, there were unique cultural and governmental factors in China that were involved. For example, the consequence of not complying with government restrictions could be administrative detention. On the other hand, the degree of uncertainty and willingness to obey social norms might make the quarantine more effective. We did not collected data to support this, which is a limitation of the study, and a promising potential topic to study in the future.

### Implications

This study provides the first recorded evidence for the psychological consequences of lockdown and quarantine during the COVID-19 pandemic, as well as a practical psychological basis for the management of public health emergencies. Identification of high-risk populations and populations with specific needs may help develop an infrastructure of response groups in the early and middle stages of the coronavirus response. In particular, those with high levels of self-stigma may need special attention.

An appropriate mental health intervention to improve the self-perceived health status, to provide instrumental and psychological support for the ‘high risk group’, and to decrease the stigmatization and discrimination from the general public could buffer the psychological impact from the epidemic. Communities, schools, and mental health agencies should provide specific trauma-related training to teachers and counselors in advance so that they are best equipped to assist others during and in the aftermath of a pandemic (Douglas et al., 2009). Telepsychiatry may have a role in addressing this need (Ng, 2011): telephone and electronic mail may also be used to supplement traditional psychotherapy, such as cognitive behavioral therapy and mindfulness therapy (Ho et al., 2020), which may minimize the need for clinicians to travel to distant, inaccessible, or remote locations. Telepsychiatry makes it possible for clinicians to access psychiatric expertise and may also potentially lessen the burden of psychiatric volunteer surge and the resultant chaos in the affected area. In addition, digital psychological programs, such as self-affirmation (Cohen, Garcia, Purdie-Vaughns, Apfel, and Brzustoski, 2009) and self-distancing (Fresco *et al*. 2007; Wang, Lippke, Miao, & Gan, 2019), are easily digitalized and have proven to be effective at reducing the impact of traumatic stress. These programs can provide effective and pertinent help to those experiencing the negative effects of the epidemic and provide the basis for developing and implementing a psychological plan during major public health emergencies. Low intensity online interventions can minimize the obstacles of time and space, as well as reflect the feasibility and effectiveness of online intervention, so that individuals can receive professional, low-intensity psychological intervention at home, thereby improving the scientific literacy of the whole population and bringing about considerable social benefits. Due to evolution in the way people access information, the use smartphones to shape individual behavior is of great significance. In addition to low-intensity online interventions, dissemination of health information related to COVID-19 via smartphones to the general public is also an important means of improving scientific literacy (Tran et al., 2020).
